# Dynamical Graph Theory Networks Methods for the Analysis of Sparse Functional Connectivity Networks and for Determining Pinning Observability in Brain Networks

**DOI:** 10.3389/fncom.2017.00087

**Published:** 2017-10-05

**Authors:** Anke Meyer-Bäse, Rodney G. Roberts, Ignacio A. Illan, Uwe Meyer-Bäse, Marc Lobbes, Andreas Stadlbauer, Katja Pinker-Domenig

**Affiliations:** ^1^Department of Scientific Computing, Florida State University, Tallahassee, FL, United States; ^2^Department of Radiology and Nuclear Medicine, Maastricht University Medical Center, Maastricht, Netherlands; ^3^Department of Electrical and Computer Engineering, Florida State University, Tallahassee, FL, United States; ^4^Department of Signal Theory and Communications, University of Granada, Granada, Spain; ^5^Department of Neurosurgery, University of Erlangen-Nürnberg, Erlangen, Germany; ^6^Memorial Sloan-Kettering Cancer Center, New York, NY, United States

**Keywords:** neurodegenerative disease, singular perturbations, area aggregation, multi-time-scale brain network, neural network, synchronization, pinning observability

## Abstract

Neuroimaging in combination with graph theory has been successful in analyzing the functional connectome. However almost all analysis are performed based on static graph theory. The derived quantitative graph measures can only describe a snap shot of the disease over time. Neurodegenerative disease evolution is poorly understood and treatment strategies are consequently only of limited efficiency. Fusing modern dynamic graph network theory techniques and modeling strategies at different time scales with pinning observability of complex brain networks will lay the foundation for a transformational paradigm in neurodegnerative diseases research regarding disease evolution at the patient level, treatment response evaluation and revealing some central mechanism in a network that drives alterations in these diseases. We model and analyze brain networks as two-time scale sparse dynamic graph networks with hubs (clusters) representing the fast sub-system and the interconnections between hubs the slow sub-system. Alterations in brain function as seen in dementia can be dynamically modeled by determining the clusters in which disturbance inputs have entered and the impact they have on the large-scale dementia dynamic system. Observing a small fraction of specific nodes in dementia networks such that the others can be recovered is accomplished by the novel concept of pinning observability. In addition, how to control this complex network seems to be crucial in understanding the progressive abnormal neural circuits in many neurodegenerative diseases. Detecting the controlling regions in the networks, which serve as key nodes to control the aberrant dynamics of the networks to a desired state and thus influence the progressive abnormal behavior, will have a huge impact in understanding and developing therapeutic solutions and also will provide useful information about the trajectory of the disease. In this paper, we present the theoretical framework and derive the necessary conditions for (1) area aggregation and time-scale modeling in brain networks and for (2) pinning observability of nodes in dynamic graph networks. Simulation examples are given to illustrate the theoretical concepts.

## 1. Introduction

Novel mathematical paradigms such as graph theoretical techniques can capture the brain connectivity and its topology (Fornito et al., 2012; Giessing and Thiel, 2012; Zeng et al., 2012). New descriptors of complex networks are able to quantify induced changes in topology or network organization or serve as theory-driven biomarkers to be used in disease prediction at the level of the individual subject.

While most graph networks applied to brain research are static graph networks and can not capture the dynamical processes governing the time evolution of neurodegenerative diseases, a new paradigm in brain research—dynamical graph networks—is required to advance this field and overcome the obstacles posed by static graph theory in terms of disease prediction and evolution, and its associated connectivity changes.

Biologically-relevant brain networks represent complex large-scale dynamical systems capturing the interaction of a large number of subsystems. Standard analysis methods and control strategies become very difficult and novel techniques need to be developed to (1) analyze the dynamical behavior of these large-scale systems or (2) to observe their states.

To address the first issue of how to control these networks, we need to employ a model simplification resulting in a model of lower complexity, that is easier to handle, and that will provide a simplified synthesis procedure for design problems at a reduced computational complexity. Balanced truncation is known as a popular method for model reduction since it is relatively simple and the quality of the reduced model is guaranteed. The interpretation of most balancing techniques is based on the concept of past and future energy. The most important contribution was the balancing for stable minimal linear systems (Moore, 1981). It is based on a state—space point of view of employing the well—known observability and controllability Gramians and related to the past input energy (controllability) and future input energy (observability). The idea behind transforming a system into balanced form is to easily detect and remove a state component of the initial system to obtain a reduced–order model. The importance of a component is based on Hankel singular values which determine if the output energy of a certain component is small and thus difficult to observe and if the input energy to reach this state is large. While for linear systems finding a balancing coordinate transformation via solutions of the controllability and observability Lyapunov equations is quite easy, for nonlinear systems these equations are almost impossible to solve and thus balancing becomes in general not a simple task (Lall et al., 2002; Scherpen, 1993). These techniques have been applied to the analysis of gene regulatory networks (Meyer-Bäse, 2008), however they are not quite efficient in terms of model reduction for large-scale networks since they involve the computationally expensive operation of matrix factorization. In addition, this method does not preserve the connection structure between subsystems and erases the neurological connection between the subsystem state variables. Therefore, a network topology-preserving mechanism to provide model reduction is required for large-scale networks. We will present an area aggregation and time-scale modeling for sparse brain networks with densely interconnected hubs and externally sparse interconnections between these hubs (Tahmassebi et al., 2017). In Biyik and Arcak (2008) it was shown that the neurons in the hubs synchronize on the fast time-scale and as aggregated neurons determine the slow dynamics of the neural network. We derive a simplified dynamic two-time scale representation for brain connectivity networks assuming linear connections between the nodes. The eigenvalues of the state matrices of the slow and fast system will provide important information about the dynamic evolution of brain connectivity networks at different stages of neurodegenerative diseases. The structural parameters of these networks expressed by the node and area parameter will unveil changes in the sparsity patterns in the course of the disease associated with the disease.

The second issue of observability of neural states can be achieved over synchronization. Synchronization plays an important role in the analysis of neural networks in neurodegenerative diseases. For many neurodegenerative diseases it is very important to obtain some information about some neural states in order to recover the others. This new concept of “pinning observability,” first proposed in Yu et al. (2014), refers to observing a small number of neurons such that the states of the other neurons can be recovered. Differently from the concept of pinning controllability (Chen et al., 2007; Liu et al., 2011; Song and Cao, 2010; Tang et al., 2012), the dynamics of the neurons can be heterogeneous. This property is extremely appealing to brain connectivity networks where we aim to obtain insight into the dynamics of several regions by observing only a few of them. We will derive for these networks a general criterion for synchronization and then some decoupled conditions for pinning observability taking into account their heterogeneous architecture.

In the present paper, we will present a reduced-model approximation over time for large-scale brain networks and derive pinning observability conditions for competitive neural networks and illustrate in an example the theoretical analysis.

## 2. Reduced-model approximation over time

Many brain connectivity networks exhibit a heterogeneous structure with densely linked nodes in an area but with sparse connections between these areas. The network is viewed as an interconnected graph with links between the areas which are viewed as nodes in the graph. Thus, the architecture can be described concisely by two main parameters (Chow and Kokotovic, 1985): the node parameter *d* and the area parameter δ. The node parameter is given as

(1) $$\begin{array}{l} d=\frac{c^{E}}{c^{I}}\ll 1 \end{array}$$

where *c*^*E*^ is the number of external links of the node with the largest number of external links to nodes outside its area and *c*^*I*^ is the number of internal links of the node with the smallest number of internal links to nodes inside its area. *d* needs to be a small number. The area parameter is given as

(2) $$\begin{array}{l} \delta =\frac{\gamma^{E}}{mc^{I}}\ll 1 \end{array}$$

where γ^*E*^ is the number of external links of the area with the largest number of external links and *m* is the minimal number of nodes found in an area.

While the node and area parameters describe the static behavior and the sparsity pattern of the network, a more interesting approach is to analyze the dynamical behavior of such a network. In order to obtain a reduced-model approximation we view the brain connectivity graph as a structured representation with dense areas (clusters) and sparse interconnections between these areas (Biyik and Arcak, 2008). The time behavior of networks with densely linked neurons in an area but with sparsely connected areas can be described dynamically by a two-time scale system where the neurons within the same area synchronize on the fast time-scale because the dense internal links allow the neural potential in the same area to quickly reach an equilibrium. During the fast time scale the exchange with the other areas is slow because of the sparsity of the links and this becomes significant only over a longer period thus leading to a slow time-scale. This coupled dynamics leads to a reduced-order model describing the long-term behavior of the overall network.

The dynamical formulation will be that the activation states within a group synchronize with each other and achieve a reference group velocity υ(*t*):

(3) $$\begin{array}{l} \underset{t\to \infty }{\lim}|x_{i}(t)-x_{j}(t)|=0,\;i,j=1,\ldots ,N\\ \;\underset{t\to \infty }{\lim}|{\dot{x}}_{i}-\upsilon (t)|=0,\;i=1,\ldots ,N \end{array}$$

The synaptic connections *d*_*ik*_ are defined as

(4) $$\begin{array}{l} d_{ik}=\{\begin{array}{lll} 1, & : & \text{if the i-th node is the positive end of the k-th link}\\ -1, & : & \text{if the i-th node is the negative end of the k-th link}\\ 0 & : & \text{otherwise} \end{array} \end{array}$$

We assume having a neural network of *N* nodes and *M* total links yielding thus a *N* × *M* incidence matrix *D* describing this neural network. The formulated objectives in Equation (3) are achieved by the following network architecture

(5) $$\begin{array}{l} {\dot{x}}_{i}=-\sum_{k=1}^{M}d_{ik}f_{k}(\theta_{k})+\upsilon (t),\;i=1,\cdots ,N \end{array}$$

where the difference variable θ_*k*_ is the difference between the positive and negative ends of k-th link, i.e.,

(6) $$\begin{array}{l} \theta_{k}:=\sum_{l=1}^{N}d_{lk}x_{l}=\{\begin{array}{lll} x_{i}-x_{j} & : & \text{if i is the positive end,}\\ x_{j}-x_{i} & : & \text{if j is the positive end}, \end{array} \end{array}$$

and *f*_*k*_(θ_*k*_) being uniformly monotone and fulfilling the sector condition *f*_*k*_(θ_*k*_)θ_*k*_ > 0 for all θ_*k*_ ∈ *R*.

In matrix representation this can be expressed as

(7) $$\begin{array}{l} \dot{x}=-Df\left(D^{T}x\right)+1_{N}\upsilon (t) \end{array}$$

with $f\left(\theta \right)={\left[f_{1}\left(\theta_{1}\right),\cdots \;,f_{M}\left(\theta_{M}\right)\right]}^{T}$ and 1_*N*_ being the *N*-vector of ones.

We reorder the incidence matrix *D* as

(8) $$\begin{array}{l} D=\left[D_{I}|D_{E}\right] \end{array}$$

such that $D_{I}=\text{diag}\left(D_{1}^{I},\ldots ,D_{r}^{I}\right)$ correspond to the *r* areas.

We assume that we have an *N*-node network with *r* internally dense regions but sparsely connected. Area α has *m*_α_ neurons with α = 1, 2, ⋯, *r* and the vector $x^{\alpha }={\left[x_{1}^{\alpha }\cdots x_{m_{\alpha }}^{\alpha }\right]}^{T}$ contains all neural activities in area α. Then we define the slow variable (Biyik and Arcak, 2008; Chow and Kokotovic, 1985) representing an aggregate region as

(9) $$\begin{array}{l} y_{\alpha }:=\sum_{i=1}^{m_{\alpha }}\frac{x_{i}^{\alpha }}{m_{\alpha }}=\frac{1}{m_{\alpha }}u_{\alpha }^{T}x^{\alpha }, \end{array}$$

i.e., *y*_α_ is the average of the components of *x*^α^, and in matrix representation, we obtain

(10) $$\begin{array}{l} y=M_{a}^{-1}U^{T}x \end{array}$$

with *M*_*a*_ = diag(*m*_1_, *m*_2_, ⋯, *m*_*r*_) and *U* = diag(*u*_1_, *u*_2_, ⋯, *u*_*r*_) where each *u*_α_ = 1_*m*_α__ is an *m*_α_-vector of 1's.

The fast variable *z*_α_ is given as the transformation of the differences between the activation of the neurons in the same region (Biyik and Arcak, 2008; Chow and Kokotovic, 1985)

(11) $$\begin{array}{l} z_{\alpha }=Q_{\alpha }x^{\alpha } \end{array}$$

where the (*m*_α_ − 1) × *m*_α_ matrix *Q*_α_ is a difference matrix. In Chow and Kokotovic (1985) *Q*_α_ is given by

(12) $$\begin{array}{l} Q_{\alpha }=\left(\begin{array}{ccccc} -1 & 1 & 0 & \cdots & 0\\ -1 & 0 & 1 & \cdots & 0\\ \vdots & \vdots & \vdots & \ddots & \vdots \\ -1 & 0 & 0 & \cdots & 1 \end{array}\right) \end{array}$$

while in Biyik and Arcak (2008) it is chosen to have orthonormal rows with the vector 1_*N*_ as a null vector:

(13) $$\begin{array}{l} Q_{\alpha }=\left(\begin{array}{ccccc} -1+(n-1)v & 1-\upsilon & -\upsilon & \cdots & -\upsilon \\ -1+(n-1)v & -\upsilon & 1-\upsilon & \cdots & -\upsilon \\ \vdots & \vdots & \cdots & \ddots & \vdots \\ -1+(n-1)v & -\upsilon & \cdots & -\upsilon & 1-\upsilon \end{array}\right) \end{array}$$

where *n* = *m*_α_ and $\upsilon =\left(n-\sqrt{n}\right)/\left(n\left(n-1\right)\right)$. In this work we will use the latter version of *Q*_α_ given in (Equation 13). This has the advantage that the pseudoinverse of *Q*_α_ is simply $Q_{\alpha }^{T}$.

In matrix form we have the fast *z* (*N* − *r*)-vector with $z={\left[z_{1}^{T}z_{2}^{T}\cdots z_{r}^{T}\right]}^{T}$

(14) $$\begin{array}{l} z\;=\;Qx \end{array}$$

with *Q* = diag(*Q*_1_, *Q*_2_, ⋯, *Q*_*r*_) being an (*N* − *r*) × *N* block diagonal matrix.

We have thus defined a new transformation of the original neural activation *X* into a slow and fast variable (Biyik and Arcak, 2008)

(15) $$\begin{array}{l} \left(\begin{array}{c} y\\ z \end{array}\right)=\left(\begin{array}{c} M_{a}^{-1}U^{T}\\ Q \end{array}\right)x \end{array}$$

Similarly to the matrix *Q* describing the connections' differences between the nodes in one area, we introduce the matrix *R*, an (*r* − 1) × *r* matrix also with orthonormal rows and with null vector 1_*r*_, that models the interarea dynamics

(16) $$\begin{array}{l} \tilde{y}=Ry \end{array}$$

where $\tilde{y}$ is the area difference variable. For further computations, we define a new matrix *C* as $C=RM_{a}^{-1}R^{T}$.

By splitting the nonlinear vector *f*(θ) from Equation (7) into two components and *U*^*T*^*D* and *QD* we obtain the block diagonal matrices representations

(17) $$\begin{array}{l} U^{T}D=\left(\begin{array}{c} 0|\Sigma \end{array}\right),f=\left(\begin{array}{c} f_{I}\\ f_{E} \end{array}\right),QD=\left(\begin{array}{c} A|B \end{array}\right) \end{array}$$

where $\Sigma =U^{T}D_{E}$, *A* is the matrix of the internal links over all areas and *B* corresponds to the external links. This yields to a new singular perturbed model

(18) $$\begin{array}{l} {\dot{\tilde{y}}}_{i}=-CR\Sigma f_{E}(\Sigma^{T}R^{T}\tilde{y}+B^{T}z)\\ \;\dot{z}=-Af_{I}(A^{T}z)-Bf_{E}(\Sigma^{T}R^{T}\tilde{y}+B^{T}z) \end{array}$$

The above model (Equation 18) applies to large-scale brain networks for sufficiently small network parameters δ and *d*. The theoretical results from Biyik and Arcak (2008) state that the neurons in the same region synchronize in fast time-scale leading to a substitute aggregate neuron in the slow time-scale.

Since most brain connectivity graph networks are modeled as linear systems, we can derive a reduced model approximation over time. We assume linear connections for the brain network and replace the nonlinearity *f* by the identity function and set υ(*t*) = 0. Then $\dot{x}$ = *Kx* = (*K*^*I*^ + *K*^*E*^)*x* with *K*^*E*^ being the external and *K*^*I*^ being the internal connection matrix.

The new linear singular perturbed system is given as

(19) $$\begin{array}{l} \left(\begin{array}{c} \dot{y}\\ \dot{z} \end{array}\right)=\left(\begin{array}{cc} \tilde{A_{11}} & \tilde{A_{12}}\\ \tilde{A_{21}} & \tilde{A_{22}} \end{array}\right)\cdot \left(\begin{array}{c} y\\ z \end{array}\right) \end{array}$$

where

(20) $$\begin{array}{l} \left(\begin{array}{cc} \tilde{A_{11}} & \tilde{A_{12}}\\ \tilde{A_{21}} & \tilde{A_{22}} \end{array}\right)=\left(\begin{array}{cc} GK^{E}U & GK^{E}Q^{T}\\ QK^{E}U & Q(K^{I}+K^{E})Q^{T} \end{array}\right) \end{array}$$

and $G=M_{a}^{-1}U^{T}$. Note that in Equation (20) we chose *Q* using the *Q*_α_ matrices given in Equation (13). If we had chosen to use (Equation 12) instead, the rows of *Q* would not have been orthonormal and the *Q*^*T*^ in $\tilde{A_{12}}$ and $\tilde{A_{21}}$ would be replaced with *Q*^+^ = *Q*^*T*^(*QQT*)^−1^.

We can determine the time-scale model by defining the fast and slow time-scales

(21) $$\begin{array}{l} t_{f}=c^{I}t\text{ and }t_{s}=\delta t_{f} \end{array}$$

and rescaling the matrices *A*_*ij*_ as

(22) $$\begin{array}{l} A_{11}=\frac{\tilde{A_{11}}}{c^{I}\delta }\;A_{12}=\frac{\tilde{A_{12}}}{c^{I}\delta }\\ A_{21}=\frac{\tilde{A_{21}}}{c^{I}d}\;A_{22}=\frac{\tilde{A_{22}}}{c^{I}} \end{array}$$

leading to a new system

(23) $$\begin{array}{l} \frac{dy}{dt_{s}}=A_{11}y+A_{12}z\\ \frac{\delta dz}{dt_{s}}=dA_{21}y+A_{22}z \end{array}$$

The above results are summarized in the theorem proven in Chow and Kokotovic (1985).

**Theorem 1**. Chow and Kokotovic (1985) *There are* δ^*^ and d^*^ *such that for all* 0 < δ ≤ δ^*^, 0 < *d* ≤ d^*^ *the system in Equation (23) has *r* slow eigenvalues and *n* − *r* fast eigenvalues. The fast and slow subsystems are given as*

(24) $$\begin{array}{l} \frac{dy_{s}}{dt_{s}}=(A_{11}-dA_{12}A_{22}^{-1}A_{21})y_{s}=A_{0}y_{s},\;y_{s}(0)=y(0)\\ \frac{dz_{f}}{dt_{f}}=A_{22}z_{f},\;z_{f}(0)=z(0)+dA_{22}^{-1}A_{21}y(0). \end{array}$$

In Chow and Kokotovic (1985) a simplified formulation of the slow system was presented, the so-called aggregate system, given as

(25) $$\begin{array}{l} M_{a}\frac{dy_{s}}{dt}=K_{a}y_{s} \end{array}$$

with $K_{a}=U^{T}K_{E}U$.

We illustrate the time-scale separation properties and the aggregation procedure first on a general example and then on a structural and functional connectivity network from healthy and dementia patients. The reduced-order model will deliver important dynamic parameters derived from the state matrices of the slow and fast subsystem that are different for healthy and dementia subjects. In addition, it determines sparsity parameters based on the area and node parameters which are different at each specific stage of the disease.

### 2.1. Examples

**Figure 1:**
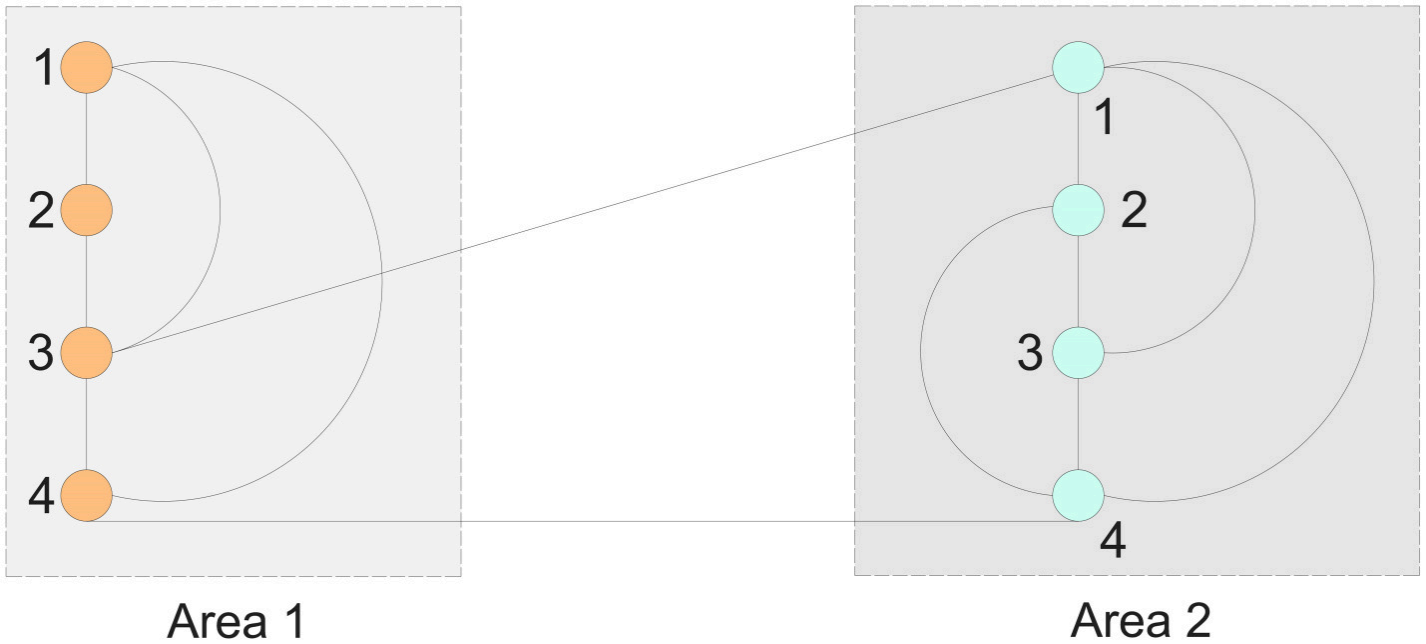
An 8-node, 2-area network.

**Example 1**. Consider an 8-node brain network that is partitioned into two areas as shown in Figure 1.

The connection matrix for Figure 1 are

(26) $$\begin{array}{l} K_{1}^{I}=\left(\begin{array}{cccc} -3 & 1 & 1 & 1\\ 1 & -2 & 1 & 0\\ 1 & 1 & -3 & 1\\ 1 & 0 & 1 & -2 \end{array}\right),K_{2}^{I}=\left(\begin{array}{cccc} -3 & 1 & 1 & 1\\ 1 & -3 & 1 & 1\\ 1 & 1 & -3 & 1\\ 1 & 1 & 1 & -3 \end{array}\right), \end{array}$$

(27) $$\begin{array}{l} K_{12}^{E}=\left(\begin{array}{cccc} 0 & 0 & 0 & 0\\ 0 & 0 & 0 & 0\\ 1 & 0 & 0 & 0\\ 0 & 0 & 0 & 1 \end{array}\right)={\left(K_{21}^{E}\right)}^{T}, \end{array}$$

(28) $$\begin{array}{l} K_{11}^{E}=\text{diag}\left(0,0,-1,-1\right),\text{ and }K_{22}^{E}=\text{diag}(-1,0,0,-1). \end{array}$$

We have *c*^*I*^ = 2, *c*^*E*^ = 1, *d* = 0.5, γ^*E*^ = 2, δ = 0.25. Further we have for the slow subsystem according to the Equation (24)

(29) $$\begin{array}{l} \frac{dy_{s}}{dt_{s}}=A_{0}y_{s} \end{array}$$

with $A_{0}=\left(\begin{array}{cc} -0.7634 & 0.7634\\ 0.7634 & -0.7634 \end{array}\right)$ and having the following two eigenvalues 0 and −1.5267. These two eigenvalues are close to those two of 0 and −2 of the matrix *A*_11_. Thus we could show that the system can be in the long-term correctly approximated by the slow subsystem.

**Example 2**. We apply the theoretical results on functional (FDG-PET) and structural (MRI) connectivity graphs (Ortiz et al., 2015) for control (CN), mild cognitive impairment (MCI) and Alzheimer's disease (AD) subjects. For the structural data, the connections in the graph show the inter-regional covariation of gray matter volumes in different areas while in the case of functional data, the connections do not show the correlation in activity but in the glucose uptake between different regions. (Ortiz et al., 2015) considered only 42 out of the 116 from the AAL in the frontal, parietal, occipital and temporal lobes. The nodes in the graphs represent the regions while the links show if a connection is existing between these regions or not.

**Figure 2:**
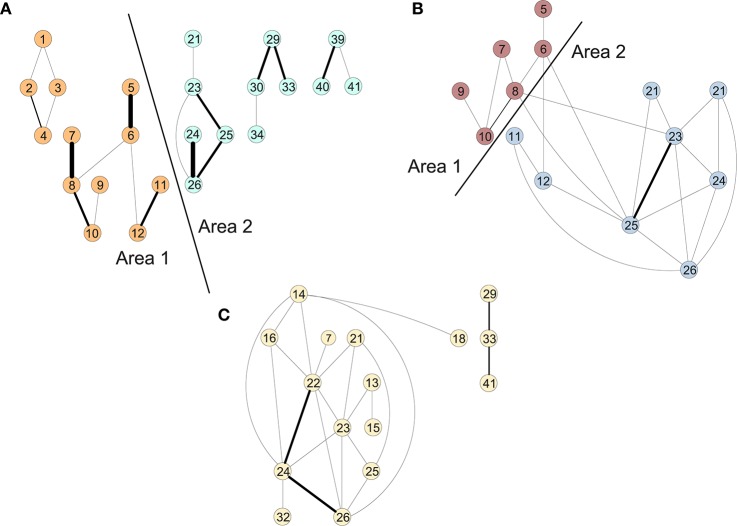
Areas of the connectivity graph for functional data for **(A)** controls, **(B)** MCI, and **(C)** AD.

Figure 2 shows the clusters found on the functional data for (A) controls, (B) MCI and (3) AD (Ortiz et al., 2015). We perform a time-scale modeling and area aggregation with two main areas on the three functional networks from Figure 2. For CN and MCI, we can apply Theorem 1, however for AD we are not able to obtain an area aggregation since the conditions in Theorem 1 are not satisfied.

The results of the in-depth dynamical analysis are shown in Table 1. The controls show smaller node and area parameters than the MCIs. But most importantly, both the exact as well as the rigid aggregate model as shown in Equation (25) show smaller eigenvalues for the controls than the MCIs.

**Table 1:** Area aggregation parameters and time-scale modeling for functional connectivity graphs.

**Subject**	**Node parameter d**	**Area parameter δ**	**Slow λ exact system**	**Slow λ rigid aggregate system**
CN	$d_{ave}=\frac{1}{5}$	$\delta =\frac{1}{5}$	λ_*i*_ = {0, −2}	λ_*i*_ = {0, −13/40}
MCI	$d_{ave}=\frac{2}{3}$	$\delta =\frac{2}{3}$	λ_*i*_ = {0, −8}	λ_*i*_ = {0, −7/6}
AD	–	–	–	–

**Figure 3:**
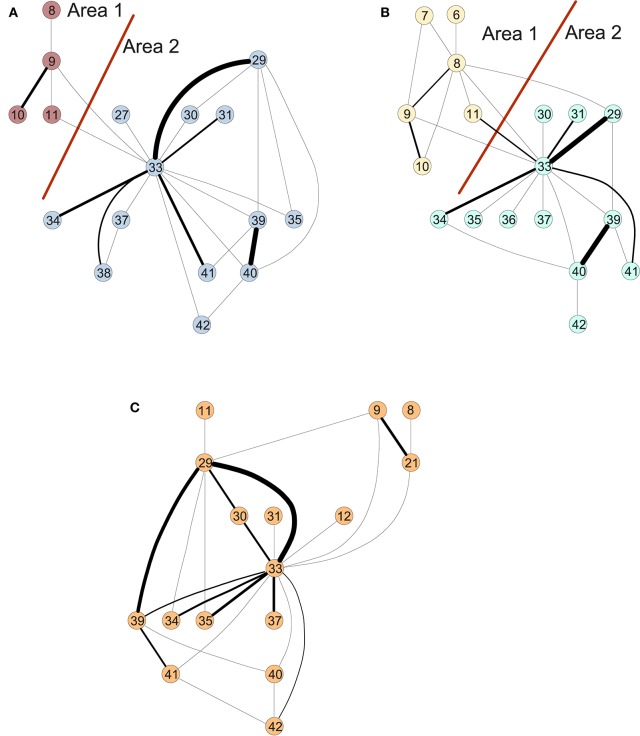
Areas of the connectivity graph for structural data for **(A)** controls, **(B)** MCI, and **(C)** AD.

Similarly, we perform a time-scale modeling and area aggregation with two main areas on the three structural networks from Figure 3. For CN and MCI, we can apply Theorem 1, however for AD we are not able to obtain an area aggregation again because the conditions are not met.

Figure 3 shows the clusters found on the structural data for (A) controls, (B) MCI and (3) AD (Ortiz et al., 2015). The results of the in-depth dynamical analysis are shown in Table 2. Similarly, the controls show smaller area parameters than the MCIs and smaller eigenvalues. However, the node parameter is larger in the case of controls.

**Table 2:** Area aggregation parameters and time-scale modeling for structural connectivity graphs.

**Subject**	**Node parameter d**	**Area parameter δ**	**Slow λ exact system**	**Slow λ rigid aggregate system**
CN	$d_{ave}=\frac{2}{3}$	$\delta =\frac{1}{2}$	λ_*i*_ = {0, −4}	λ_*i*_ = {0, −17/26}
MCI	$d_{ave}=\frac{4}{7}$	$\delta =\frac{2}{3}$	λ_*i*_ = {0, −8}	λ_*i*_ = {0, −1}
AD	–	–	–	–

While the results obtained through static graph analysis revealed the loss of strong connections in AD and MCI compared to CN, the dynamic graph analysis reveals different slow modes between MCI and CN. The CN have smaller eigenvalues than the MCI for both functional and structural data and those eigenvalues remain operative. The contribution of the larger eigenvalues over time decreases quickly. The range of the eigenvalues for each subject represents an important biomarker for disease prediction. It is worth noting that an area aggregation is not possible for the ADs in both structural and functional networks since the conditions of Theorem 1 are not fulfilled. By providing an area and node parameter, we are able to add to the dynamic biomarkers additional static graph descriptors. The reduced-order time scale modeling provides a change in the sparsity pattern for MCI and AD patients compared to the CN, and shows higher values for the node and area parameters.

It is worth mentioning that the slow or aggregate variable represents in Markov chains the probability for a Markov process to be in a group of states.

## 3. Pinning observability

Brain connectivity networks have lots of nodes and connections between them, and it is effectively impossible to observe the states of all nodes such that the network can be recovered by reaching synchronization between the original network and a reconstructed network. Pinning observability is introduced as a new technique to reduce the number of observable nodes, and at the same time to be able to recover the states of other nodes. We give a new criterion for synchronization via pinning observability for nonlinear brain networks and derive decoupled conditions for pinning observability for brain connectivity networks.

We consider in the following the general neural network equation describing the temporal evolution of the neural activation states for the *i*th neuron of an *N*–neuron network:

(30) $$\begin{array}{l} {\dot{x}}_{i}=A_{i}x_{i}+\sum_{j=1}^{n}{\tilde{d}}_{ij}\bar{f}(x_{j})\;i=1,\ldots ,N \end{array}$$

where $x_{i}={\left(x_{i1}\left(t\right),\cdots \;,x_{in}\left(t\right)\right)}^{T}\in R^{n}$ is the state vector of node *i*, $A_{i}\in R^{n\times n}$ is a matrix, and $\bar{f}\left(x_{i}\right)$ is the neuron's output. ${\tilde{d}}_{ij}$ represents a synaptic connection parameter between the *i*th neuron and the *j*th neuron and is defined as the matrix $\tilde{D}=\left({\tilde{d}}_{ij}\right)$.

Pinning observability is applied only to a small number *l* of nodes and thus we obtain a pinning observable network supposing these first *l* nodes are selected:

(31) $$\begin{array}{l} {\dot{\tilde{x}}}_{i}=A_{i}{\tilde{x}}_{i}+\sum_{j=1}^{n}{\tilde{d}}_{ij}\bar{f}({\tilde{x}}_{j})+u_{i}\;i=1,\ldots ,N \end{array}$$

where

(32) $$\begin{array}{l} g_{i}(x_{j})=\sum_{j=1}^{N}{\tilde{d}}_{ij}\bar{f}(x_{j}) \end{array}$$

and

(33) $$\begin{array}{l} u_{i}=-d_{i}({\tilde{x}}_{i}(t)-x_{i}(t))\;1,\cdots ,l \end{array}$$

are *n*-dimensional linear feedback controllers with the control gains *d*_*i*_ > 0, *i* = 1, ⋯, *l* and *d*_*i*_ = 0 for *i* = *l* + 1, ⋯, *N*.

By subtracting the pinned system Equation (31) and the original system Equation (30), we obtain the error dynamical system

(34) $$\begin{array}{l} {\dot{e}}_{i}(t)=A_{i}e_{i}(t)+g_{i}(\tilde{x_{1}}(t),\cdots ,\tilde{x_{N}}(t))-g_{i}(x_{1}(t),\cdots ,x_{N}(t)) \end{array}$$

and the error signal

(35) $$\begin{array}{l} e_{i}(t)=\tilde{x_{i}}(t)-x_{i}(t). \end{array}$$

The following assumptions and lemmas are needed to obtain the main result of the paper.

*Assumption A* Assume a scale-free graph network and let $\tilde{D}={\left({\tilde{d}}_{ij}\right)}_{N\times N}$ be the coupling configuration matrix. Then there exists a symmetric matrix Γ such that

(36) $$\begin{array}{l} \sum_{i=1}^{N}{\left(\hat{x_{i}}-\hat{w_{i}}\right)}^{T}(g_{i}(\hat{x_{1}}(t),\cdots ,\hat{x_{N}}(T))-g_{i}(\hat{w_{1}}(t),\cdots ,\hat{w_{N}}(t)))\\ \leq \sum_{i=1}^{N}\sum_{j=1}^{N}l_{ij}{\left(\hat{x_{i}}-\hat{w_{i}}\right)}^{T}\Gamma \left(\hat{x_{j}}-\hat{w_{j}}\right). \end{array}$$

The following two lemmas will be useful.

**Lemma 4**. *For any vectors x, y* ∈ *R^N^ and positive definite matrix G* ∈ *R*^*N*×*N*^, *the following matrix inequality holds*

(37) $$\begin{array}{l} 2x^{T}y\leq x^{T}Gx+y^{T}G^{-1}y. \end{array}$$

**Lemma 5**. *Schur complement* (Boyd et al., 1994). *The following linear matrix inequality (LMI)*

(38) $$\begin{array}{l} \left(\begin{array}{cc} Q(x) & S(x)\\ S{\left(x\right)}^{T} & R(x) \end{array}\right)>0 \end{array}$$

with *Q*(*x*) = *Q*(*x*)^*T*^, *R*(*x*) = *R*(*x*)^*T*^ is equivalent to either of the following equivalent conditions

(39) $$\begin{array}{l} (1)\;Q(x)>0,R(x)-S{\left(x\right)}^{T}Q{\left(x\right)}^{-1}S(x)>0\\ (2)\;R(x)>0,Q(x)-S(x)R{\left(x\right)}^{-1}S{\left(x\right)}^{T}>0 \end{array}$$

We can now state a result concerning the two networks Equations (30) and (31).

**Theorem 6**. *Under Assumption A the two networks Equations (30) and (31) are globally synchronized if the following condition is satisfied*

(40) $$\begin{array}{l} (A+A^{T})/2-D⊗I_{n}+(L⊗\Gamma +L^{T}⊗\Gamma )/2<0 \end{array}$$

where *A* = diag(*A*_1_, …, *A*_*N*_), *D* = diag(*d*_1_, …, *d*_*l*_, 0, …, 0), $\tilde{D}={\left({\tilde{d}}_{ij}\right)}_{N\times N}$ and *I*_*n*_ being an *n*-dimensional identity matrix.

*Proof*. Consider the Lyapunov function candidate

(41) $$\begin{array}{l} V(t)=\frac{1}{2}\sum_{i=1}^{N}e_{i}^{T}(t)\cdot e_{i}(t). \end{array}$$

We take the derivative of *V*(*t*) along the trajectories of Equation (34) and obtain

(42) $$\begin{array}{l} \dot{V}(t)=\sum_{i=1}^{N}e_{i}^{T}(t)\cdot \dot{e_{i}}(t)\\ \;=\sum_{i=1}^{N}e_{i}^{T}(t)(A_{i}e_{i}(t)+g_{i}(\tilde{x_{1}}(t),\cdots ,\tilde{x_{N}}(t))\\ \;-g_{i}(x_{1}(t),\cdots ,x_{N}(t))). \end{array}$$

From Assumption A we have

(43) $$\begin{array}{l} \;\sum_{i=1}^{N}e_{i}^{T}(t)\left(g_{i}(\tilde{x_{1}},\cdots \tilde{x_{N}},t)-g_{i}(x_{1},\cdots x_{N},t)\right)\\ \leq \sum_{i=1}^{N}\sum_{j=1}^{N}{\tilde{d}}_{ij}e_{i}^{T}(t)\Gamma e_{j}(t). \end{array}$$

Substituting Equation (43) into Equation (42), we have

(44) $$\begin{array}{l} \dot{V}(t)\leq \sum_{i=1}^{N}e_{i}^{T}(t)\cdot (A_{i}-d_{i}I_{n})\cdot e_{i}(t)\\ \;+\sum_{i=1}^{N}\sum_{j=1}^{N}l_{ij}e_{i}^{T}(t)\Gamma e_{j}(t)\\ \;=e^{T}(t)\left((A+A^{T})/2-D⊗I_{n}+\tilde{D}⊗\Gamma \right)e(t). \end{array}$$

As shown in Yu et al. (2014), it is possible to identify the number of nodes that can be observed. The following corollary gives a condition to check for each node whether it can be controlled or not without involving the other nodes.

Synchronized if there exists a constant *c* > 0 such that the following condition is satisfied

**Corollary 7**. *Under the Assumption A, the two networks Equations (30) and (31) are globally synchronized if there exists a constant c* > 0 *such that the following condition is satisfied*

(45) $$\begin{array}{l} (A_{i}+A_{i}^{T})/2-d_{i}I_{n}+{\tilde{d}}_{ii}\Gamma +\frac{1}{2}c_{i}\sum_{j=1,j\neq i}^{N}|{\tilde{d}}_{ij}|\Gamma \Gamma^{T}\\ \;+\frac{1}{2}\sum_{j=1,i\neq j}^{N}\frac{1}{c_{j}}|{\tilde{d}}_{ji}|I_{n}<0 \end{array}$$

The proof is based on using the same Lyapunov function as in the above Theorem.

Thus we are able to give a much simpler condition with this fixed constant *c*: if *d*_*i*_ = 0 and the condition given in the below equation is satisfied for a node *i*, then the node may not be controlled. Otherwise, if this condition is not satisfied for node *i*, then the node can be controlled.

$$\begin{array}{l} \Xi_{i}(c)=\left(\begin{array}{cc} (A_{i}+A_{i}^{T})/2-d_{i}I_{n}+l_{ii}I_{n}+\frac{c}{2}\sum_{j=1,j\neq i}^{N}l_{ij}I_{n} & \sqrt{\sum_{j=1,i\neq j}^{N}|l_{ij}|}I_{n}\\ \sqrt{\sum_{j=1,i\neq j}^{N}|l_{ij}|}I_{n} & -2cI_{n} \end{array}\right)<0,\\ \;i=1,2,\cdots ,N. \end{array}$$

The above equation shows a simple modality to find the few nodes that have to be controlled such that synchronization is reached.

The following examples are given to show the application of the theory to a simple neural network. We illustrate the concept of pinning observability first on a general example describing a network with nonlinear coupling and then on a network processing olphactoric stimuli with linear coupling. Both examples employ a simplified condition Equation 46 for pinning observability developed in the above theoretical framework.

### 3.1. Examples

**Example 3**. In the following we give a numerical example to elucidate the theoretical results in pinning observability.

The simplest example consists of three neurons. Let *N* = 3, *a*_1_ = 2, *a*_2_ = 1.6, *a*_3_ = 0.5, *c* = 1 $\tilde{D}=\left(\begin{array}{ccc} 0.7 & -0.2 & -0.1\\ -0.2 & 0.5 & -0.3\\ -0.1 & -0.3 & 0.8 \end{array}\right)$, and let the nonlinearity *f* be a sigmoid function with *K* = 1.

The condition to be tested for each neuron is given by Theorem 6.

In case the condition is satisfied for a fixed parameter *c* and *d*_*i*_ = 0, then the neuron *i* can not be controlled. Node 3 is the one that needs to be controlled and *d*_1_ = 1.5.

**Figure 4:**
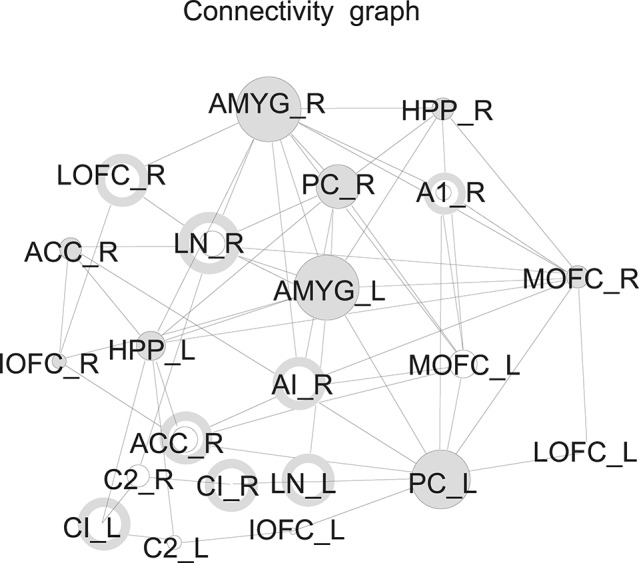
Nodes that can be controlled in the connectivity graph during olphactoric stimulation marked as a donut (Nigri et al., 2013).

**Example 4**. The processing of olphactoric stimuli was shown to involve a network of cortical and subcortical regions (Nigri et al., 2013). The resulting connectivity graph in fMRI studies shows a small number of hubs in this network suggesting that they have a predominant role in information gathering. Applying the theory of pinning observability on this networks reveals that there are few nodes in the graphs that can be controlled, and that these nodes are surprisingly not hubs but have sparse connections. Figure 4 shows the distribution of these controlling nodes in the olphactoric network.

## 4. Discussion

In this paper, we introduced some novel dynamic graph theory techniques to the analysis of the dynamical behavior of brain connectivity networks. We applied the new concepts of time-scale modeling for sparse networks and pinning observability on brain networks of heterogeneous architecture. We considered graphs with densely linked nodes in an area but with sparse connections between these areas. We have shown that the nodes in a dense area synchronize on the fast time-scale while the dense areas become aggregate nodes on the slow time scale. For the time scale modeling, we have derived new local models that describe the fast and slow dynamics of brain connectivity networks assuming linear connections between the nodes. This new paradigms provides us with important disease descriptors showing changes over the disease trajectory such as the modes of the dynamic system and the sparsity patterns.

Observing a small number of nodes in brain connectivity network and recovering the states of the others is of major interest in a large-scale network. This was achieved through pinning observability. We formulated a new criterion for synchronization for nonlinear brain networks and derived decoupled simplified conditions for determining the small number of observable states in the network. Examples are given to elucidate the theoretical results for both new concepts.

While static graph theory shows the changes in graph measures at certain points in time and the differences between disease and normal control, the derived results may have important implication for understanding and controlling the evolution of neurodegenerative diseases that may further lead to better therapeutic interventions. Thus by describing the dynamic of the aggregate areas and the resulting time-scale modeling and determining the observable nodes in a brain network, a new research avenue is opened that allows more detailed study of the differences between disease and healthy groups and which provides a wider variety of characteristic parameters over time for those groups.

## Author contributions

All authors listed have made a substantial, direct and intellectual contribution to the work, and approved it for publication. AM: manuscript writing, research and revision, RR, IAI, UM, ML, AS, and KP: research and revision.

### Conflict of interest statement

The authors declare that the research was conducted in the absence of any commercial or financial relationships that could be construed as a potential conflict of interest.
